# A multicenter survey of asymptomatic cryptococcal antigenemia among patients with advanced HIV disease in Nigeria

**DOI:** 10.1371/journal.pgph.0001313

**Published:** 2023-01-31

**Authors:** Rita O. Oladele, Alexander M. Jordan, Joy U. Okaa, Iriagbonse I. Osaigbovo, Shuwaram A. Shettima, Nathan Y. Shehu, Adeyinka A. Davies, Yahaya Mohammed, Mary A. Alex-Wele, Garba Iliyasu, Jude C. Nwaokenye, Samuel A. Fayemiwo, Ubong A. Udoh, Titilola Gbajabiamila, David W. Denning, Tom M. Chiller

**Affiliations:** 1 Department of Medical Microbiology and Parasitology, College of Medicine University of Lagos, Lagos, Nigeria; 2 Medical Mycology Society of Nigeria, Lagos, Nigeria; 3 Mycotic Diseases Branch, Division of Foodborne, Waterborne, and Environmental Diseases, Centers for Disease Control, Atlanta, Georgia, United States of America; 4 Department of Pharmaceutical Microbiology and Biotechnology, Nnamdi Azikiwe University, Awka, Nigeria; 5 Department of Medical Microbiology, University of Benin Teaching Hospital, Benin City, Nigeria; 6 Department of Medical Microbiology, Parasitology and immunology, Modibbo Adama University Teaching Hospital, Yola, Nigeria; 7 West African Centre for Emerging Infectious Disease, Jos University Teaching Hospital, Jos, Nigeria; 8 Department of Medical Microbiology, Olabisi Onabanjo University Teaching Hospital, Sagamu, Nigeria; 9 Department of Medical Microbiology and Parasitology, Faculty of Basic Clinical Sciences, College of Health Sciences, Usmanu Danfodiyo University, Sokoto, Nigeria; 10 Department of Medical Microbiology and Parasitology, University of Port Harcourt Teaching Hospital, Port Harcourt, Nigeria; 11 Infectious Disease Unit, Department of Medicine, College of Health Sciences, Bayero University, Kano, Nigeria; 12 Department of Medical Microbiology and Parasitology, University College Hospital, Ibadan, Nigeria; 13 Department of Medical Microbiology and Parasitology, University of Calabar, Calabar, Nigeria; 14 Clinical Sciences Department, Nigerian Institute of Medical research, Lagos, Nigeria; 15 Division of Infection, Immunity and Respiratory Medicine, Faculty of Biology, Medicine and Health, Manchester Academic Health Science Centre, University of Manchester, Manchester, United Kingdom; University of Colorado Anschutz Medical Campus: University of Colorado - Anschutz Medical Campus, UNITED STATES

## Abstract

As of 2018, cryptococcal antigen (CrAg) screening in patients with advanced human immunodeficiency virus (HIV) disease (AHD) was not routinely implemented in Nigeria despite being recommended in the national HIV treatment guidelines. Our aim was to determine the prevalence and risk factors for asymptomatic cryptococcal antigenemia in adult people living with HIV (PLHIV) in Nigeria to advocate for the implementation of routine CrAg screening. A descriptive cross-sectional study and CrAg screening of consecutive adult PLHIV with CD4 counts ≤200 cells/*μ*L was conducted from April 2018 to April 2019 at HIV clinics in eleven tertiary hospitals spread across Nigeria’s six geopolitical regions. Prevalence of asymptomatic cryptococcal antigenemia was estimated by facility and geopolitical zone. Logistic regression was conducted to identify risk factors for cryptococcal antigenemia. In total, 1,114 patients with AHD were screened. The overall prevalence of asymptomatic cryptococcal antigenemia was 3.9% with wide variation across facilities (range: 0/75 [0%]– 15/122 [12.3%]) and geopolitical zones (range: 0/75 [0%]–19/279 [6.8%]). Prevalence of antigenemia was highest in the South-West (19/279 [6.8%]) and lowest in the North-East (0/75 [0%]). Prevalence was 5.2% (26/512) and 3.2% (18/561) in patients with CD4<100 and CD4 of 101–200, respectively. Of all patients with antigenemia, 50% were on antiretroviral therapy (ART) at the time of having a positive CrAg test. In adjusted analysis, cryptococcal antigenemia was significantly less in patients on ART and patients who had completed any formal education. The survey showed a high overall burden of cryptococcal antigenemia in Nigeria, with variable prevalence across geopolitical regions. We provided valuable evidence for implementing routine CrAg screening of AHD patients in Nigeria which has commenced in selected centres.

## Introduction

Over the last decade there has been a significant increase in access to antiretroviral therapy (ART) for people living with HIV (PLHIV) in resource limited settings [1]. However, an estimated 30% of ART naïve patients in sub-Saharan Africa still present to care with advanced HIV disease (AHD), defined as CD4<200 cells/μL or clinical stage 3–4 illness. These patients are at high risk of morbidity and death from opportunistic infections such as tuberculosis (TB), pneumocystis pneumonia, and cryptococcal meningitis (CM). CM is a significant cause of global morbidity and mortality for PLHIV, especially in resource-limited settings. HIV-associated CM occurs primarily in patients with AHD. The most recent analysis of the global burden of CM estimates a global incidence of 223,100 cases annually, with 73% of these cases occurring in sub-Saharan Africa [2]. The fatality rate of CM is high in resource limited settings. The average 1-year mortality from cryptococcal meningitis in these settings is estimated to be 70% (range: 59–81%) for those accessing healthcare and 100% for those not accessing healthcare [2]. Although PLHIV who are ART-naïve are at the highest risk for CM, the disease can also occur in the ART-experienced, especially those who have interrupted ART or are experiencing virological failure. An analysis from Botswana showed that the incidence of CM observed in 2013–2014 was similar to pre-ART era incidence observed in South Africa, despite dramatically improved ART coverage in Botswana [3]. A dramatic increase in the proportion of PLHIV at high risk of CM due to severely depleted immune status who are ART-experienced has also been observed in South Africa in recent years [4].

A first episode of cryptococcal antigenemia indicates disseminated infection and cryptococcal antigen (CrAg) can be detected in a patient’s blood weeks to months prior to onset of CM [5]. Routine screening of serum, plasma, or whole blood for asymptomatic CrAg in patients with AHD, coupled with pre-emptive fluconazole therapy for those with antigenemia but have not yet developed CM, has been demonstrated to reduce the incidence of CM [6]. It has also been shown to reduce overall mortality when coupled with enhanced ART adherence efforts [7]. Furthermore, CrAg screening has been shown to be cost-effective in the setting of routine implementation by national HIV programs in Botswana [8], South Africa [9], and Uganda [10]. For this reason, routine CrAg screening of patients with AHD is now recommended in national HIV guidelines of more than 20 African countries, including Nigeria [11]. Nigeria is estimated to have one of the highest absolute numbers of HIV-associated CM cases in the world [2]. Since 2016, the Nigerian National guidelines for HIV prevention, treatment, and care recommend CrAg screening in ART naïve adults with CD4< 200 cells/mm^3^ followed by pre-emptive antifungal therapy to prevent CM in patients with antigenemia in whom concurrent CM is ruled out [12]. However, no routine implementation of CrAg screening and pre-emptive treatment was taking place in Nigeria at the time of this study. In view of the non-implementation of CrAg screening in Nigeria, the authors conducted a study to assess the prevalence of antigenemia in selected HIV clinics in Nigeria; and to identify demographic and clinical factors associated with cryptococcal antigenemia. The data generated guided advocacy and implementation of CrAg screening and pre-emptive therapy for the AHD population in selected centres in Nigeria.

## Materials and methods

### Study design and location

This descriptive cross-sectional study was conducted across eleven ART clinics located in all six geopolitical zones of Nigeria; (North Central- Jos; North East–Yola; North West- Sokoto, Kano; South South- Calabar, Port Harcourt, Benin city; South East–Anambra; South West–Ibadan, Lagos, Sagamu). The sites within the zones were selected mostly based on confirmation of a high burden of AHD from the national database but also because of the availability of suitably qualified site investigators with an established working relationship. Institutional ethical approval was obtained at each site before commencement of the study.

### Study population

The studied population consisted of adult (18 years and above) patients with AHD patients who had CD4 count less than 200 cells/μL or clinical stage 3–4 disease. CD4 count testing is done per routine at the participating sites, with all ART naïve patients eligible for baseline CD4 while CD4 monitoring is done every 6 months for patients on ART. Patients in these two categories were eligible for the study if their CD4 count was less than 200 cells per microlitre. Patients who were currently pregnant or breastfeeding, had previously been treated for cryptococcosis, or who had clinical liver disease (presence of jaundice, abdominal swelling, chronic fatigue and other symptoms in the presence of deranged liver function tests) were excluded from the study. All eligible patients were screened for CrAg regardless of ART status.

Eligible patients were enrolled consecutively until the end of the study period.

### Sample size

We selected a minimum sample size of 120 participants per site based on the minimum number recommended to establish a reference interval using the the non-parametric percentile method [13]. This is because of the wide geographical variations in disease prevalence typically seen in Nigeria and also because some sites had no prior data on CrAg.

### Data collection

Demographic data and clinical history were collected using a pre-tested, semi-structured questionnaire. Blood samples were obtained and transported to the laboratory at each site where CD4 and CrAg testing were conducted on patients’ serum samples. CrAg testing was performed by laboratory personnel previously trained to conduct the test using the CrAg lateral flow assay (LFA) (IMMY Inc., Norman, OK, USA) per manufacturer’s instructions. The same lot of testing kits was sent to all the sites, however to rule out false positives or negatives, duplicate samples were shipped to the Principal Investigator’s laboratory where they were retested. There was very good correlation between the results. However, a couple of samples had prozone reactions which was discovered by serial dilutions. These samples were recorded as positive for cryptococcal antigen. Relevant clinical and laboratory data was obtained from the enrolled patients’ records. CrAg screening was conducted from April 2018 –April 2019.

All final clinical and treatment initiation decisions (ART and antifungal) for enrolled patients were made at the discretion of the clinicians at the participating facilities based on the standard of care at the time of the pilot. However, we recommended that patients who tested positive for CrAg and were asymptomatic for cryptococcal meningitis be commenced on Fluconazole 800mg daily for 2 weeks, then deescalated to 400mg daily for 8–10 weeks, followed by 200mg daily until immune reconstitution according to Nigerian Ministry of Health HIV treatment guidelines [12]. Patients who were symptomatic for CM were recommended to have lumbar puncture done if there were no contraindications and with positive cerebrospinal fluid (CSF) CrAg results prescribed treatment for cryptococcal meningitis per Nigerian guidelines. Patient symptomology, CSF CrAg results, CM treatment status, and clinical outcomes were not captured as part of our study.

### Ethical considerations

Ethical approval was obtained from ethical committees at all participating enrolment sites prior to implementation of patient enrolment and screening activities. (Names of ethical review committees and the study approval numbers are listed in S1 File). Informed written consent was obtained from each study participant after adequate explanation of the study and its objectives. Participants specimen and data were stripped of personal identifiers and given unique codes. Data collection tools were stored in a secure cabinet. All participant data were entered into a private, secure computer with access limited to investigators. Test results were kept confidential and disclosed only to the patient and their healthcare provider.

### Data analysis

Analyses were performed using SAS 9.4 (SAS Institute Inc., Cary, NC, USA). A 5% significance level was used throughout the study unless otherwise specified. Chi-squared and Fisher’s exact tests were used to compare proportions between groups and Wilcoxon rank sum tests were used to compare continuous variables. Bivariable and multivariable logistic regression was conducted to identify factors associated with higher odds of cryptococcal antigenemia. Any variable with a p-value ≤0.2 in bivariable analysis was considered in the initial adjusted model. The final model included all variables for which adjustment was necessary to account for potential confounding (assessed by impacts on effect size and precision when removing variables in backward model selection). Collinearity and correlation were assessed prior to conducting regression analysis.

## Results

### Enrolment and Sociodemographic characteristics

From April 2018 to April 2019, 1,114 patients with advanced HIV disease (AHD) were enrolled and screened for CrAg (Table 1). Of those enrolled, 402 (36.1%) were ART-naïve and 712 (63.9%) were receiving ART. Patients receiving ART were designated as ART-experienced irrespective of the length of time they had received ART. The proportion of enrolled patients who were ART-naïve varied significantly by facility (p-value: <0.0001). Only one facility (Shagamu; in SW geopolitical zone) enrolled more ART-naïve patients than ART-experienced patients. The median age of enrolled patients was 39 (IQR: 33–47). The age of ART-experienced patients was significantly higher than ART naïve patients (median age of 40 vs 38: p-value <0.001). The majority (59.9%) of enrolled patients were female. Female patients were significantly more likely to be ART-experienced compared to male patients (p-value: 0.02). The vast majority (90.7%) of patients reported being either employed or retired at the time of enrolment. Enrolled patients had largely (88.5%) received some formal education (defined as primary school certificate or higher).

**Table 1:** Demographic and clinical variables of patients screened for CrAg.

	Overall N (%)	ART-Naïve (%)	ART–Experienced (%)	p-value^a^
	N = 1,114	n = 402	n = 712	
Median age (IQR)	39 (33–47)	38 (30–45)	40 (34–48)	<0.001
**Facility**				
Anambra	92 (8.3)	45 (11.2)	47 (6.6)	<0.0001
Benin City	125 (11.2)	5 (1.2)	120 (16.9)	
Ibadan	122 (11.0)	39 (9.7)	83 (11.7)	
Jos	138 (12.4)	63 (15.6)	75 (10.5)	
Kano	100 (9.0)	48 (11.9)	52 (7.3)	
Lagos	32 (2.9)	7 (1.7)	25 (3.5)	
Calabar	83 (7.5)	12 (3.0)	71 (10.0)	
Port Harcourt	112 (10.1)	44 (10.9)	68 (9.6)	
Shagamu	125 (11.2)	79 (19.7)	46 (6.5)	
Sokoto	110 (9.9)	38 (9.5)	72 (10.1)	
Yola	75 (6.7)	22 (5.5)	53 (7.4)	
**Sex**				
Female	664 (59.6)	226 (56.2)	438 (61.5)	0.017
Male	444 (39.9)	171 (42.5)	273 (38.3)	
Unknown	6 (0.5)	2 (0.5)	4 (0.6)	
**Employment Status**				
Employed or retired	1004 (90.1)	363 (90.3)	641 (90.0)	0.92
Unemployed	103 (9.2)	37 (9.2)	66 (9.3)	
Unknown	7 (0.6)	2 (0.5)	5 (0.7)	
Marital Status				
Married	573 (51.4)	201 (50.0)	372 (52.2)	0.54
Single	269 (24.1)	103 (25.6)	166 (23.3)	
Divorced	90 (8.21	38 (9.5)	52 (7.3	
Widowed	159 (14.3)	52 (12.9)	107 (15.0)	
Unknown	23 (2.1)	8 (2.0)	15 (2.1)	
**Education Status**				
No formal education	127 (11.4)	43 (10.7)	84 (11.8)	0.68
Any formal education	979 (87.9)	357 (88.8)	622 (87.4)	
Unknown	8 (0.7)	2 (0.5)	6 (0.8)	
**CD4**				
Median CD4 (IQR)	107 (52–161)	102 (52–159)	111 (52–162)	0.74
**Cryptococcal antigenemia**				
Yes	44 (3.9)	22 (5.s)	22 (3.1)	0.05
No	1070 (96.1)	380 (94.5)	690 (96.9	

^a^ P-values were used to compare features of ART_naive and ART-experienced patients; chi-square or Fisher tests for proportions were used to compare categorical variables, and Wilcoxon signed-rank test was used to compare continuous variables.

### Clinical presentation

CD4 results were available for 1095 (98.3%) patients, with 19 (1.7%) patients tested in the absence of a CD4 result due to having a clinical stage 3–4 illness. Median CD4 count was 107 (IQR: 52–161) (Table 1). Of those enrolled (N = 1114), 1073 (96.3%) had a CD4≤200 cells/mm^3^, 512 (46.0%) had a CD4≤100 cells/mm^3^, 561 (51.2%) had a CD4 of 101–200, and 22 (1.9%) had a CD4>200 cells/mm^3^ and were tested due to clinical stage 3–4 illness. CD4 count did not differ significantly by ART status at the time of enrolment (p-value: 0.73).

### Cryptococcal antigenemia

**Fig 1:**
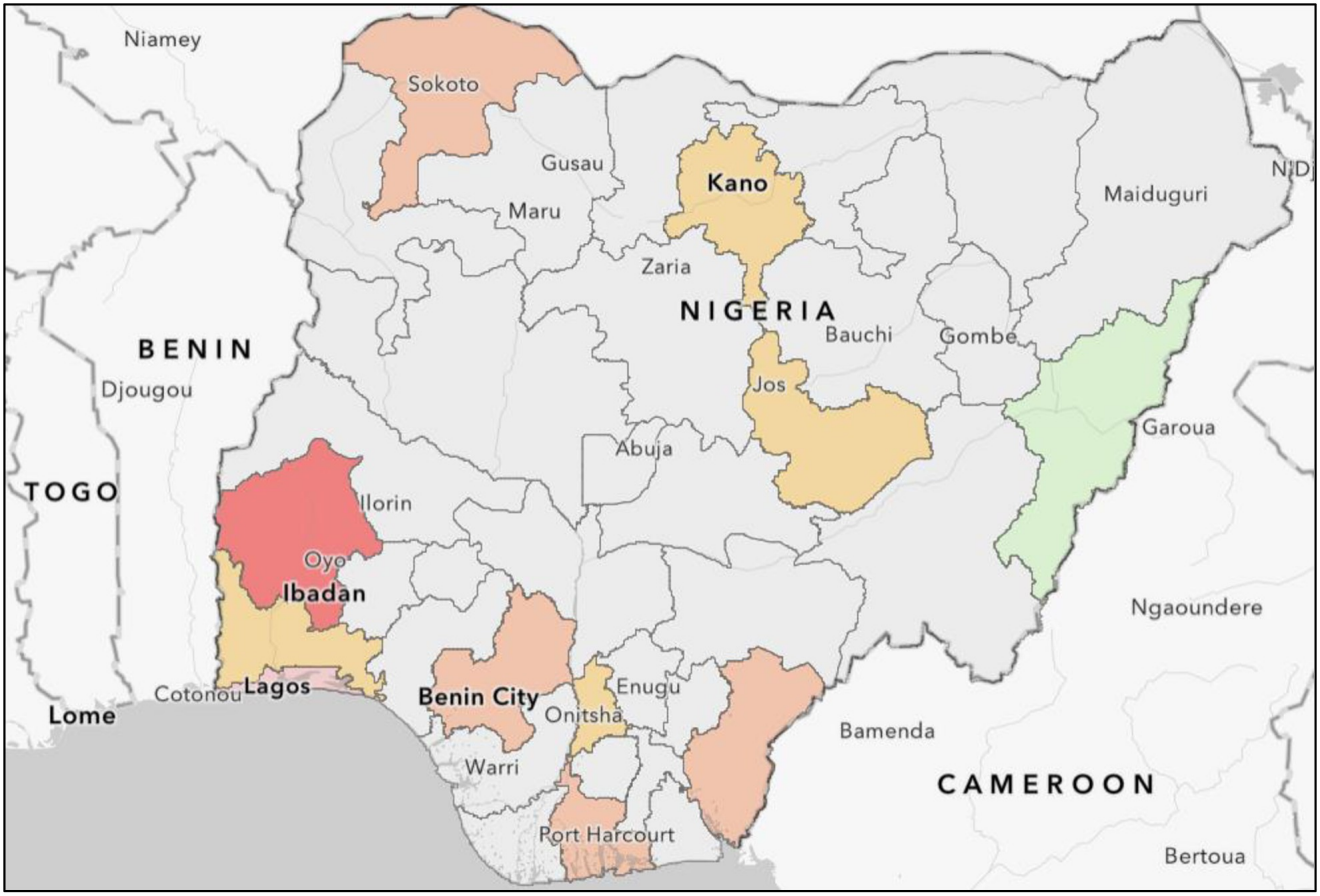
Map of Nigeria showing prevalence of cryptococcal antigenaemia at the various sites. Red–Greater than 10%; Pink–>5 to 10%; Dark orange–>3 to 5%; Light orange–>0 to 3%; Green– 0%; Grey–No data (Map created using ArcGIS, basemap available at https://basemaps.arcgis.com/arcgis/rest/services/World_Basemap_v2/VectorTileServer).

The overall prevalence of antigenemia was 3.9% (44 / 1114) (95% C.I: 2.9–5.3) (Table 2). The prevalence varied across facilities (range: 0/75 [0%]– 15/122 [12.3%]) and geopolitical zones (range: 0/75 [0%]–19/279 [6.8%]) (Table 2). CrAg prevalence was highest in the facilities in the South-West (19/279 [6.8%]) and North-West (7/210 [3.3%]) zones. CrAg prevalence was lowest in North-East zone (0/75 [0%]), which included only 1 study facility (Table 2; Fig 1). Total prevalence was 5.2% (26/512) and 3.2% (18/561) in patients with CD4<100 and CD4 of 101–200, respectively. None of the 41 patients with CD4>200 cells/mm^3^ or with clinical stage 3–4 illness without a CD4 result tested CrAg positive. Of all patients with antigenemia, 22 (50%) were receiving ART at the time of screening. No measured factors were significantly associated with the odds of cryptococcal antigenemia in bivariable analysis. However, in adjusted analysis, patients who had previously received ART for any amount of time (aOR: 0.52, 95% CI: 0.28–0.96) and patients who had completed any formal education (aOR: 0.42, 95% CI: 0.20–0.92) had significantly lower odds of cryptococcal antigenemia (Table 3).

**Table 2:** Cryptococcal antigenemia by Geopolitical zone and health facility.

Geopolitical Zone	No. Enrolled	ART Naive (%)	ART Experienced (%)	CD4≤100 (%)^a^	CD4≤200 (%)^a^	CrAg-positive (%)	Percentage Contribution to overall burden of CrAg	95% Confidence Interval
***North-West***	**210**	**86 (41.0)**	**124 (59.0)**	**82 (39.0)**	**196 (93.3)**	**7 (3.3)**		**1.3–6.8**
Kano	100	48 (48.0)	52 (52.0)	50 (50)	100 (100)	2 (2.0)	4.5	0.2–7.0
Sokoto	110	38 (34.5)	72 (65.5)	32 (29.1)	96 (87.3)	5 (4.5)	11.4	1.5–10.3
***North-East***	**75**	**22 (29.3)**	**53 (70.7)**	**39 (52.0)**	**73 (97.3)**	**0 (0)**		**-**
Yola	75	22 (29.3)	53 (70.7)	39 (52.0)	73 (97.3)	0 (-)	0	-
***North-Central***	**138**	**63 (45.7)**	**75 (54.3)**	**52 (37.7)**	**125 (90.6)**	**3 (2.2)**		**0.5–6.2**
Jos	138	63 (45.7)	75 (54.3)	52 (37.7)	125 (90.6)	3 (2.2)	6.8	0.5–6.2
***South-West***	**279**	**125 (44.8)**	**154 (55.2)**	**133 (47.7)**	**269 (96.4)**	**19 (6.8)**		**4.2–10.4**
Ibadan	122	39 (32.0)	83 (68.0)	49 (40.2)	116 (95.1)	15 (12.3)	34.1	7.1–19.5
Lagos	32	7 (21.9)	25 (78.1)	18 (56.3)	30 (93.8)	2 (6.3)	4.5	0.8–20.8
Shagamu	125	79 (63.2)	46 (36.8)	66 (52.8)	123 (98.4)	2 (1.6)	4.5	0.2–5.7
***South-East***	**92**	**45 (48.9)**	**47 (51.1)**	**53 (57.6)**	**92 (100)**	**2 (2.2)**		**0.3–7.6**
Anambra	92	45 (48.9)	47 (51.1)	53 (57.6)	92 (100)	2 (2.2)	4.5	0.3–7.6
***South-South***	**320**	**61 (19.1)**	**259 (80.9)**	**153 (47.8)**	**318 (99.4)**	**13 (4.1)**		**2.2–6.9**
Benin City	125	5 (4.0)	120 (96.0)	63 (50.4)	124 (99.2)	5 (4.0)	11.4	1.3–9.1
Calabar	83	12 (14.5)	71 (85.5)	47 (56.6)	82 (98.8)	4 (4.8)	9.1	1.3–11.9
Port Harcourt	112	44 (39.3)	68 (60.7)	43 (38.4)	112 (100)	4 (3.6)	9.1	1.0–8.9
**Total**	**1114**	**402 (36.1)**	**712 (63.9)**	**512 (46.0)**	**1073 (96.3)**	**44 (3.9)**	**100**	**2.9–5.3**

Note:

^a^19 enrolled patients did not have CD4 counts and were enrolled due to stage 3 or 4 illness; ART status, CD4 count category and CrAg positivity are expressed as percentages of number enrolled (row percentages)

**Table 3:** Risk factors for cryptococcal antigenemia.

Factor	CrAg-tested N = 1114 (%)	CrAg-positive N = 44 (%)	CrAg-negative N = 1072 (%)	Bivariate Analysis				Multivariate Analysis			
				OR	95% Confidence Interval		P-value	aOR	95% Confidence Interval		P-value
					Lower	Upper			Lower	Upper	
Age (Categorized)											
18–24	60 (5.4)	4 (6.7)	56 (93.3)	Ref	-	-	-	-	-	-	-
25–39	486 (43.6)	13 (2.7)	473 (97.3)	0.39	0.12	1.22	0.10	-	-	-	-
40–59	494 (44.3)	21 (4.3)	473 (95.8)	0.62	0.21	1.88	0.40	-	-	-	-
≥60	49 (4.4)	4 (8.2)	45 (91.8)	1.24	0.30	5.25	0.77	-	-	-	-
Unknown	25 (2.2)	2 (8.0)	23 (92.0)	-	-	-	-	-	-	-	-
Gender											
Female	664 (59.6)	31 (4.7)	633 (95.3)	Ref	-	-	-	-	-	-	-
Male	444 (39.9)	13 (2.9)	431 (97.1)	0.62	0.32	1.19	0.15	0.64	0.33	1.25	0.19
Unknown	6 (0.5)	0 (0)	6 (100)	-	-	-	-	-	-	-	-
Employment Status											
Employed or retired	1004 (90.1)	40 (4.0)	964 (96.0)	Ref	-	-	-	-	-	-	-
Unemployed	103 (9.3)	4 (3.9)	99 (96.1)	1.03	0.36	2.93	0.96	-	-	-	-
Unknown	7 (0.6)	0 (0)	7 (100)	-	-	-	-	-	-	-	-
Marital Status											
Married	573 (51.4)	21 (3.7)	552 (96.3)	Ref	-	-	-	-	-	-	-
Single	269 (24.2)	11 (4.1)	258 (95.9)	1.12	0.53	2.36	0.76	-	-	-	-
Divorced	90 (8.1)	2 (2.2)	88 (97.8)	0.60	0.14	2.59	0.49	-	-	-	-
Widowed	159 (14.3)	10 (6.3)	149 (93.7)	1.76	0.81	3.83	0.15	-	-	-	-
Unknown	23 (2.1)	0 (0)	23 (100)	-	-	-	-	-	-	-	-
Education status											
No formal education	127 (11.4)	9 (7.1)	118 (92.9)	Ref	-	-	-	Ref	-	-	-
Any formal education	979 (87.9)	35 (3.6)	944 (96.4)	0.49	0.23	1.04	0.06	0.42	0.20	0.92	0.03
Unknown	8 (0.7)	0 (0)	8 (100)	-	-	-	-	-	-	-	-
CD4 level											
CD4≤100	512 (46.0)	26 (5.1)	486 (94.9)	Ref	-	-	-	Ref	-	-	-
CD4 101–200	561 (50.4)	18 (3.2)	543 (96.8)	0.62	0.34	1.14	0.13	0.62	0.33	1.15	0.13
CD4≥200	22 (1.9)	0 (0)	22 (100)	-	-	-	-	-	-	-	-
Unknown	19 (1.7)	0 (0)	19 (100)	-	-	-	-	-	-	-	-
ART status											
ART-naïve	402 (36.1)	22 (5.5)	378 (94.3)	Ref	-	-	-	Ref	-	-	-
ART-experienced	712 (63.9)	22 (3.1)	694 (97.7)	0.55	0.30	1.00	0.05	0.52	0.28	0.96	0.04

## Discussion

A number of African countries, including Nigeria, have adopted a 200 cells/mm^3^ CD4 cell count threshold for their national cryptococcal screening guidelines [11, 12]. To our knowledge, this is the first multicenter study to prospectively assess the prevalence of cryptococcal antigenemia amongst AHD patients in Nigeria at the CD4 threshold of 200 cells/μL.

The overall prevalence of cryptococcal antigenemia in this study (3.9%) fits within the range (1.7%–15.8%) of previously observed CrAg prevalence reported from countries in sub-Saharan Africa [2]. It is considerably higher than the 2.3% prevalence reported from a large, retrospective, cross-sectional study conducted by Ezeanolue et al across three geographic regions in Nigeria using archived blood samples collected from 2004 to 2014 [14]. The retrospective approach adopted by Ezeanolue and colleagues and the time frame in which their study was conducted does not allow an adequate snapshot of the current prevalence of cryptococcal antigenaemia given that ART access must have varied considerably within the study period. The higher prevalence observed in our study, while not directly comparable to the previous study, does indicate that there was still a relatively high burden of cryptococcal antigenemia (and therefore high risk of CM) in PLHIV with CD4<200 cells/μL in Nigeria as of 2019, despite expanded access to ART in Nigeria in the intervening period.

The Ezeanolue *et al* study found a significantly higher prevalence (4.4%) in the South-East compared to the South-West (1.4%) and the combined North-West and North-Central (0.5%) geopolitical zones. Other single-center studies have demonstrated CrAg prevalence of 1.6% (irrespective of CD4 count) and 12% (at CD4<200 cells/μL) at tertiary hospitals in Kano (North-West) and Benin City (South-South), respectively [15, 16]. The results of our survey provide further evidence that the prevalence of cryptococcal antigenemia may vary substantially by location and region in Nigeria. However, our study found that the prevalence of antigenemia was highest in the South-West (6.8%), driven by a particularly high prevalence in Ibadan (12.3%). The northernmost facility (Sokoto–North-West) was also found to have a notably high prevalence of antigenemia (4.5%) compared to that reported from the North-West by Ezeanolue *et al*. Other recent studies have also indicated a high prevalence of antigenemia in Sokoto [17]. The observed variation in prevalence of cryptococcal antigenemia across studies could be influenced by numerous factors, including unidentified clinical factors and differential environmental conditions favoring growth of *Cryptococcus spp*. Although the environmental niches are not comprehensively defined, *Cryptococcus spp* is known to be associated with certain types of soil, flora, and fauna [18]. A recent study conducted in Zambia found that the diversity of *Cryptococcus spp*. isolated from environmental samples was associated with distinct ecological niches across the country [19]. Previous research has also suggested that the virulence of *Cryptococcus spp*. varies by lineage and species [20], suggesting that the risk of developing HIV-associated cryptococcosis could also vary with local ecological factors [21]. The observed prevalence in our study may have been affected to some extent by differential enrollment across facilities. Despite the majority of facilities meeting their target sample sizes (120), some facilities did not, with one facility in Lagos only enrolling 32 patients. Notably, one facility where no cryptococcal antigenemia was detected also enrolled a relatively low number of patients (75), due in part to challenges in the area related to the Boko Haram insurgency.

We observed that almost 60% of patients with antigenemia had a CD4 count≤100 cells/μL. However, in adjusted analysis we found that the odds of cryptococcal antigenemia in patients with CD4 of 101–200 cells/μL was not significantly lower compared to patients with CD4≤100 cells/μL (aOR: 0.62, p-value: 0.13). This is in contrast to previous studies in Nigeria which have found the risk of cryptococcal antigenemia is significantly higher in PLHIV with CD4 counts <100 cells/ μL [14, 15]. Our results indicate that the Nigerian recommendation to screen patients with CD4<200 cells/mm^3^ for CrAg is warranted and may lead to detection of a substantial number of patients with antigenemia who would be missed if screening were confined to lower CD4 thresholds.

The odds of antigenemia in this study was not significantly associated with age or gender, which is consistent with previously published results from Nigeria [14, 17]. However, the majority of patients with antigenemia were of working age and the vast majority reported being actively employed. This indicates that the patients with AHD and cryptococcal antigenemia identified in this study represent a population that is at high risk of morbidity and mortality from CM while still active and productive in the community. The protective effect of formal education observed in our analysis is a novel finding and may reflect socioeconomic factors which affect the likelihood of a patient obtaining timely and adequate HIV diagnosis and treatment. Previous evidence has shown that the direction of the association between education and HIV-infection has varied over the course of the HIV/AIDS pandemic in sub-Saharan Africa [22]. This indicates that the relationship between education level, engagement with HIV care, and cryptococcal antigenemia is a complex one, and is likely dependent on regional and local context, including the structure of the healthcare system, and the availability of and access to routine HIV services in a given country or region.

Half of the patients with antigenemia in this analysis were receiving ART at the time of their positive CrAg screening result, but with unknown ART initiation dates. This means that some of these patients could have been on ART for a short period of time if their routine baseline CD4 testing was delayed until follow-up visits post-ART initiation. However, the results of our multivariable analysis show that being on ART at the time of positive CrAg test was significantly protective against cryptococcal antigenemia. Therefore, it is likely that our estimate of the protective effect of ART against cryptococcal antigenemia is a conservative one and an even more protective effect of ART would have been observed in patients on ART for longer periods of time.

In any case, this result highlights the importance of early HIV diagnosis and prompt ART initiation, coupled with CrAg screening of those with advanced HIV and initiation of pre-emptive antifungal therapy to reduce the incidence and mortality associated with CM in adult PLHIV with cryptococcal antigenemia. Early identification of PLHIV and initiation of ART before a patient’s immune system becomes seriously compromised is still considered the most effective way to reduce the number of patients at risk for serious OIs such as CM [23]. Late HIV diagnosis is, unfortunately, still an obstacle to reducing HIV-associated mortality. A large proportion of patients initiating ART globally already have AHD, and are therefore at a higher risk of early HIV-associated mortality even after initiating treatment [1]. Furthermore, HIV-support programs in Sub-Saharan Africa have, since 2008, shifted focus from emergency response to sustainability and country ownership, policy changes which have been accompanied by substantial cuts in funding needed to access required diagnostics and medicines have resulted in increased healthcare costs for patients with AHD [24]. This may affect their ability to afford gold-standard interventions and treatment for CM and other OIs after enrollment in care. A comprehensive review by Lahuerta et al showed that there are multiple individual and system-level issues that result in patients not initiating ART early [25]. These issues include patients’ financial constraints, a lack of awareness on the part of PLHIV, overly centralized testing services which limit access to HIV testing, and human resource limitations within the health system, among others. Fortunately, there are interventions which can help to reduce morbidity and mortality from HIV-associated mortality due to CM [1]. The affordability (~2.50 USD) and accuracy (>95% sensitivity and specificity) of the CrAg LFA makes this a generally feasible approach even in resource limited setting. Despite this, at the time this study was conducted, no ART facility in Nigeria was implementing routine CrAg screening and pre-emptive fluconazole treatment as part of routine HIV care.

The study was limited by lack of data on patient symptoms, viral suppression status, length of time on ART in enrolled patients, and patient outcomes. Nevertheless, our results illustrate that a considerable proportion of people with advanced HIV in Nigeria are at high risk of CM in the absence of comprehensive CrAg screening and pre-emptive fluconazole treatment as part of routine HIV care.

As a result of the data generated from this study progress is being made in implementing routine CrAg screening and pre-emptive treatment in Nigeria. At the time of writing, the Clinton Health Access Initiative (CHAI) and Unitaid, a global health funding agency, have partnered with National AIDS and STDs Control Programme (NASCP) to support implementation of the WHO recommended package of care for AHD [26], including CrAg screening and pre-emptive fluconazole therapy in selected sites in Nigeria, with support from Medical Mycology Society of Nigeria and other organisations. This is a crucial first step if Nigeria is to realize the recently proposed global goal of ending deaths from HIV-associated CM by 2030 [27].

To conclude, this nationally-representative survey showed a high overall burden of cryptococcal antigenemia in Nigeria, with variable prevalence across geopolitical regions. The data generated provided valuable evidence for implementing routine CrAg screening of AHD patients in Nigeria which has commenced in selected centres.

## Supporting information

S1 File Ethical review committees approving this study and study approval numbers. (DOCX)

S1 Data Data supporting the study. (XLSX)
