# Supplementary material for: A multicenter survey of asymptomatic cryptococcal antigenemia among patients with advanced HIV disease in Nigeria
Source: PLOS Glob Public Health. 2023 Jan 31;3(1):e0001313. doi: 10.1371/journal.pgph.0001313 (PMC10021610; doi:10.1371/journal.pgph.0001313)
Supplement: S1 File — (DOCX) [file pgph.0001313.s001.docx]

**S1 File. Ethical Approvals for ‘A multicenter survey of cryptococcal antigenemia among advanced HIV disease patients in Nigeria’**

| **S/N** | **Name of Ethical Review Committee** | **Study Approval Number** | **Approval Date** |
| --- | --- | --- | --- |
| 1. | Usmanu Danfodiyo University Teaching Hospital (UDUTH) Health Research Ethics Committee | UDUTH/HREC/2017/No. 598 | May 22, 2017 |
| 2. | Olabisi Onabanjo University Teaching Hospital (OOUTH) Health Research Ethics Committee | OOUTH/HREC/155/2017 | October 5, 2017 |
| 3. | University of Port Harcourt Teaching Hospital (UPTH) Research Ethics Committee | UPTH/ADM/90/S.II/VOL.XI/433 | June 28, 2017 |
| 4. | University of Benin Teaching Hospital (UBTH) Ethics and Research Committee | ADM/E22/A/VOL.VII/14504 | August 4, 2017 |
| 5. | University of Ibadan (UI)/University College Hospital (UCH) Ethics Committee | UI/EC/17/0250 | August 17, 2017 |
| 6. | Chukwuemeka Odumegwu Ojukwu University Teaching Hospital (COOUTH) Ethical Committee | COOUTH/CMAC/ETH.C/VOL.1/0034 | April 23, 2018 |
| 7. | University of Calabar Teaching Hospital (UCTH) Health Research Ethics Committee | UCTH/HREC/33/530 | April 21, 2017 |
| 8. | Jos University Teaching Hospital (JUTH) Health Research Ethics Committee | JUTH/DCS/ADM/127/XXVIII/981 | July 23, 2018 |
| 9. | Federal Medical Centre, Yola Health Research Ethical Committee | FMCY/SUB/96N/T/XIII | May 4, 2017 |
| 10. | Bayero University, Kano College of Health Sciences Research Ethics Committee (CHS-REC) | BUK/CHS/REC/V1/49 | May 14, 2018 |
| 11. | Nigerian Institute of Medical Research Institutional Review Board (NIMR-IRB) | IRB/18/021 | July 5, 2018 |
